# Potential mosquito vector attraction to- and feeding preferences for pigs in Romanian backyard farms

**DOI:** 10.3389/fvets.2022.1046263

**Published:** 2023-01-04

**Authors:** Jonno Jorn Stelder, Andrei Daniel Mihalca, Ann Sofie Olesen, Lene Jung Kjær, Anette Ella Boklund, Thomas Bruun Rasmussen, Mihai Marinov, Vasile Alexe, Oana Maria Balmoş, René Bødker

**Affiliations:** ^1^Section for Animal Welfare and Disease Control, Department of Veterinary and Animal Sciences, Copenhagen University, Copenhagen, Denmark; ^2^Department of Parasitology and Parasitic Diseases, University of Agricultural Sciences and Veterinary Medicine of Cluj-Napoca, Cluj-Napoca, Romania; ^3^Department of Virus and Microbiological Special Diagnostics, Statens Serum Institut, Copenhagen, Denmark; ^4^Department of Biodiversity Conservation and Sustainable Use of Natural Resources, Danube Delta National Institute for Research and Development, Tulcea, Romania

**Keywords:** blood meal, african swine fever virus, mechanical transmission, west-nile virus, japanese encephalitis virus, insect vectors

## Abstract

**Introduction:**

Mosquitoes either biologically or mechanically transmit various vector-borne pathogens affecting pigs. Mosquito species display a wide variety of host preference, as well as host attraction and behaviours. Mosquito species attraction rates to- and feeding rates on pigs or other potential hosts, as well as the seasonal abundance of the mosquito species affects their pathogen transmission potential.

**Methods:**

We caught mosquitoes in experimental cages containing pigs situated in Romanian backyard farms. The host species of blood meals were identified with PCR and sequencing.

**Results:**

High feeding preferences for pigs were observed in *Aedes vexans* (90%), *Anopheles maculipennis* (80%) and *Culiseta annulata* (72.7%). However, due to a high abundance in the traps, *Culex pipiens/torrentium* were responsible for 37.9% of all mosquito bites on pigs in the Romanian backyards, despite low feeding rates on pigs in the cages (18.6%). We also found that other predominantly ornithophilic mosquito species, as well as mosquitoes that are already carrying a blood meal from a different (mammalian) host, were attracted to backyard pigs or their enclosure.

**Discussion:**

These results indicate that viraemic blood carrying, for instance, African swine fever virus, West-Nile virus or Japanese encephalitis virus could be introduced to these backyard pig farms and therefore cause an infection, either through subsequent feeding, via ingestion by the pig or by environmental contamination.

## 1. Introduction

Flying hematophagous insects that are known to feed on wild boar and domestic pigs have been described as vectors of a wide variety of pathogens (1–4), with some of these affecting pigs (Suidae) (5). In terms of vector-borne transmission of pathogens, two distinct mechanisms are described, namely biological- and mechanical transmission (6). With biological transmission, a pathogen is able to replicate within the body of a vector before it is delivered to a new host, whereas with mechanical transmission, the pathogen cannot replicate and is eventually digested or shed, thus requiring transmission to a new host while still infectious (6). An example of pathogens transmitted by biological vector transmission is Japanese encephalitis virus [mosquitoes (7)], while examples of pathogens transmitted by mechanical vector transmission are porcine reproductive and respiratory syndrome (8), Ross River Virus [mosquitoes (9)], *Mycoplasma suis* [mosquitoes and *Stomoxys calcitrans* (10)], and classical swine fever [mosquitoes (11), *S. calcitrans* (12), and tabanids (13)].

Mosquitoes can act as vectors between vertebrate species for pathogens of increasing societal concern. Mosquitoes infected with West-Nile virus (WNV) or Japanese encephalitis virus (JEV) after feeding on an infected bird can act as bridge-vectors to pigs (7, 14, 15), and pigs can then act as important amplification hosts for the virus (5). As different mosquito species can show varying host preferences (16), it is necessary to determine the host-preferences of each mosquito species to evaluate its transmission potential for various pathogens.

Hematophagous insects are potential mechanical vectors of African swine fever virus (ASFV) (17), and in the Baltic countries a seasonal pattern is observed in outbreaks in domestic pigs coinciding with the summer peak abundance pattern observed in many hematophagous insect species (18, 19). Various studies suggest that mechanical transmission by vectors can occur in an experimental setting (20), for instance through ingestion (21), environmental contamination (22) or subsequent blood feeding (23), and may occur in a natural setting (24, 25). Insect species frequently attracted to, and feeding on, pigs are particularly interesting when exploring the risk of introducing pathogens to domestic pig farms by contaminated insects. Other studies have indicated that ASFV contaminated trucks (26) or professional farm visitors (27) could be a source of ASFV introduction to commercial pig farms, although the exact transmission mechanism behind these two risk factors is not yet fully understood. From this, we can hypothesise that the introduction of viraemic blood into a pig farm through hematophagous insects, if carrying a sufficient viral load, could potentially cause an outbreak. It is therefore important to identify which hematophagous insect species would potentially feed on an ASFV infected pig host outside of a farm, before they could be attracted to domestic pigs and thus introduce virus into a pig farm.

Studies show that throughout the vector season in Europe (in Sweden, Italy and the Netherlands), *Culex* spp. are significantly more abundant than any other mosquito genus, particularly *Culex pipiens*, while *Aedes* spp. also takes up a significant proportion of the total number of mosquitoes throughout the year (28). Differences in overall abundance are important as, while host preferences can vary between species (29), it is not necessarily the species with the strongest preference towards a certain host that is the most relevant in terms of vector potential of a certain pathogen (14, 30).

Spread of pathogens by hematophagous insects could be influenced by differences in seasonal activity patterns, species abundance, locality, host-diversity or host-preference, insect species size, digestion as well as reproductive behaviour and strategies. We designed an experiment in Romania to quantify which species of mosquitoes are attracted to Romanian backyard pigs, which species take blood meals from these, and whether these observed feeding behaviours vary throughout the vector season. The findings from this experiment could provide more evidence for the introduction of pathogens in blood meals by flying vectors.

## 2. Methodology

**Figure 1:**
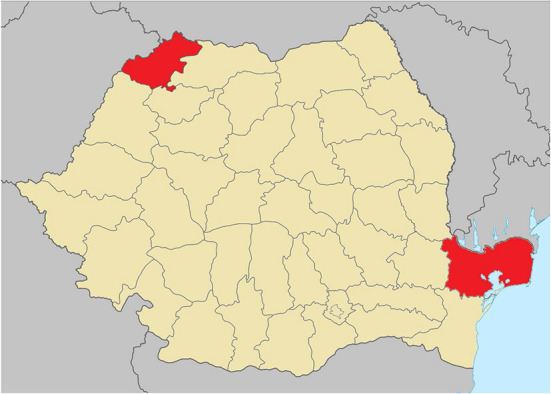
Map of Romania with Satu Mare County (locations Ambud and Odoreu) highlighted in red in the Northwest and Tulcea County (locations Somova and Salcioara) highlighted in red in the Southeast.

We selected two backyard pig farm locations in Tulcea County (Somova and Sӑlcioara, Southeast Romania, GPS coordinates: 45°11′41“N 28°38′52“E & 44°47′57“N 28°53′49“E) and another two in Satu Mare County (Ambud and Odoreu, Northwest Romania, GPS coordinates: 47°45′59“N 22°56′22“E & 47°47′54“N 22°58′59“E) (Figure 1). We selected the specific localities based on availability and suitability for our experimental purposes, as well as there being ASFV outbreaks in these regions.

### 2.1. Cages and pigs

**Figure 2:**
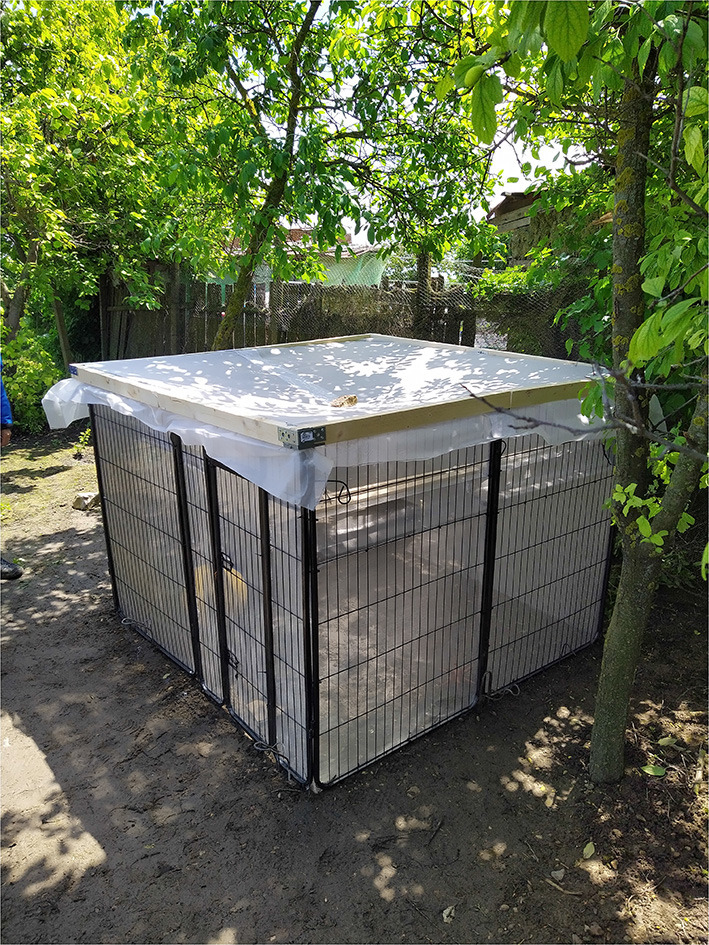
One of the pig cages used in the experiment (pigs not yet included). The entry trap slit can be seen on the front right panel. Collectors entered the cage through a small door, as can be seen on the front left panel, by carefully opening it and closing it immediately upon entry, so no insects would fly out.

Domestic pigs were kept outside in modified dog puppy cages (~120 cm height × 160 cm length × 160 cm depth) fitted with fine stainless-steel mesh (mesh size: 0.6 mm) sheets on the walls and fine mesh on the roof. For this, we obtained an animal experiment ethical permit, registration number: 216, issued 12-06-2020 by the Bioethics Commission of USAMV Cluj-Napoca, Romania. We conducted animal care and maintenance in accordance with EU legislation on animal experimentation (EU Directive 2010/63/EU). We applied an entry-trap design, which allowed flying insects to fly into the cages through a ~5 cm cage-wide slit on two sides of the cages (Figure 2). In each of the 4 locations, we kept 2 pigs weighing between 7.5 and 10 kg each at the onset of their respective sampling period.

### 2.2. BG-Pro traps

In each of the 4 locations, we also set up a BG-Pro mosquito trap (BioGents AG). We baited each of these traps with CO_2_ (0.5 kg of CO_2_ per 24 h) and BG-Lure mosquito attractants (which emit lactic- and caproic acid as well as ammonia). We placed the traps within the vicinity of the cages to catch mosquitoes continuously throughout the season, and we collected the mosquitoes once a week. These 7-day catches allowed us to quantify abundance of mosquitoes as well as correlate it to the proportion of mosquitoes with pig blood in mosquitoes collected from the pig cages.

### 2.3. Sampling periods

We carried out sampling during June to October 2021. For the cages, we aimed at 3 sampling periods of 4 weeks each, whereas the BG-Pro mosquito traps ran continuously throughout the study. For each sampling period, we replaced the pigs with new pigs, leading to 24 pigs in total during the study. The first collection period started on the 14th of June 2021 and ended on the 7th of July. In Satu Mare, the start of the second period was delayed due to logistical issues and the collection was extended beyond the original collection schedule for this period from the 16th of August until the 8th of September. In Tulcea, the second collection period started on the 1st of August and was also extended until the 8th of September to align with the collections in Satu Mare. The third collection period in both regions started on the 20th of September and ended on the 13th of October 2021.

### 2.4. Sampling procedure

We cleaned the cages once per week, after which we placed the mesh roofs on top of the cages, which indicated the start of that week's collection. We entered the cages 24 h later. Using aspirators, we collected all hematophagous insects present inside the cages and stored them at −20°C. Another 24 h later, we would repeat this process, after which we removed the roofs of the cages for the remainder of the week. For each of the four locations, we therefore had two 24 h consecutive collections of hematophagous insects from the cages per week for a total of 12 weeks.

### 2.5. Species identification

In the high containment laboratory (OIE3/4, BSL2) at Statens Serum Institut (Denmark), we identified each sample from the cages to species level using the Moskeytool identification tool (https://www.medilabsecure.com/moskeytool.html). We then visually screened each mosquito for the presence of a blood meal according to the Sella score (1–7) where Sella score 1 and 7 indicate no blood in the abdomen, and Sella score 2–6 indicates varying amounts of blood present (31).

Due to large numbers of mosquitoes collected in the BG-Pro traps, we subsampled collections when necessary and identified the mosquitoes only to genus level. We subsampled collections using a divider in a Petri dish until a single subsample contained <350 mosquitoes. We then extrapolated the results from the selected subsample to the original sample. We did not screen these mosquitoes any further or analyse them for the presence of blood meals.

### 2.6. Lab procedure

After species identification, we placed each mosquito with blood in the abdomen (Sella score between 2 and 6) individually in a 1.5 ml Eppendorf tube and added 1 ml MagNA Pure Lysis/Binding Buffer (Roche). We homogenised the samples using two 3 mm stainless steel beads (Dejay Distribution Ltd.) in a TissueLyser II (Qiagen) for 3 min at 25 Hz, after which we centrifuged the sample homogenates for 2 min at 10.000 RCF to collect supernatants for nucleic acid extraction. We purified the nucleic acids from the homogenised sample supernatants as previously described by Olesen et al. (32) and analysed for the presence of a porcine blood meal (i.e. MT-CYTB from Suidae) applying a TaqMan assay (33) using the CFX Opus Real-Time PCR System (BioRad). We determined a positive result (Suid blood present) in this qPCR by identification of the threshold cycle value (Cq) at which FAM dye emission appeared above background within 35 cycles.

Samples testing negative for porcine blood were tested using the TaqMan assay (No Cq value or Cq value above 35) for the presence of a mammalian blood meal (i.e. MT-CYTB of mammalian origin) using a Resolight (Roche) approach with primer sequences obtained from Andrejevic et al. (34), with minor modifications. The slightly modified primer sequences were 5′ GACGGCCAGTGAAACAGGATCCAACAACCC 3′ (forward) and 5′ GCTATGACCGGTGTAGTTGTCTGGGTCTCC 3′ (reverse). We performed the amplification using the CFX Opus Real-Time PCR System (Bio-Rad). We determined a positive result (mammalian blood present) in this qPCR by identification of the Cq value at which SYBR dye emission appeared above background within 30 cycles. We chose this threshold of 30 based on prior validation using templates of different mammalian, avian and invertebrate origin. During this validation, samples with Cq values above 30 were of avian and invertebrate origin.

We selected samples in which we detected a blood meal of mammalian origin, using the mammalian MT-CYB assay, for Sanger sequencing. For sequencing, we purified PCR products using the GeneJET PCR Purification Kit (Thermo Scientific) according to the manufacturer's instructions. We performed cycle sequencing of the PCR products using the Veriti™ 96-Well Thermal Cycler (Applied Biosystems). We performed the sequencing of the reverse and forward strand using 10 μM of the primers and the BigDye Terminator v. 1.1. Cycle Sequencing Kit (Applied BioSystems). For capillary electrophoresis, we purified the sequencing products using the SigmaSpin Post-Reaction columns (Sigma-Aldrich). We carried out capillary electrophoresis out using an ABI3500 Genetic Analyzer (Applied BioSystems). Ultimately, we analysed the results using Sequence Scanner Software v1.0 (Applied BioSystems) and the blood meal source identified using BLAST (https://blast.ncbi.nlm.nih.gov/Blast.cgi).

## 3. Results

In total, we identified 356 mosquitoes and 1 tabanid from the cages in the 4 locations. From the Satu Mare region, this includes 204 mosquitoes from Ambud and 96 mosquitoes from Odoreu, while from the Tulcea region this includes 38 mosquitoes and 1 tabanid samples from Sălcioara and 18 mosquito samples from Somova. Unfortunately, some of the samples from Tulcea turned unidentifiable upon arrival to the high containment laboratory, as unforeseen leakage of a number of sample containers in the same shipment necessitated use of decontamination (disinfection) upon arrival to the laboratory. This caused disinfectant to seep into some of the containers. This caused the mosquitoes inside 12 out of 20 (60%) 24 h collections from Sălcioara and 15 out of 20 (75%) 24 h collections from Somova to become unidentifiable, while it was still possible to count the numbers of mosquitoes in most collections excluding 6 collections from Somova, where we estimated the numbers visually.

**Figure 3:**
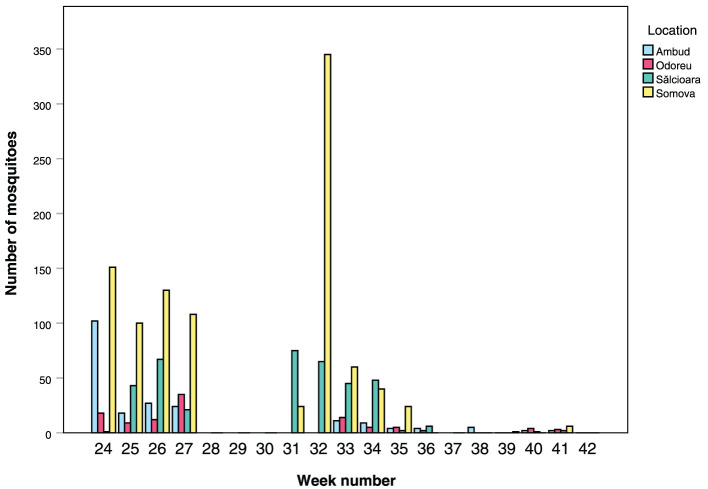
Number of mosquitoes caught inside the pig cages per week for all four locations. Note that there were no pigs inside the cages, and thus no collections, in week 28, 29, 30 (+ 31, 32 for Satu Mare's Ambud and Odoreu locations) and week 37.

From the Tulcea region (Somova and Sălcioara), and in particular Somova, we caught larger numbers of mosquitoes in the cages throughout the experiment, compared to the Satu Mare region (Ambud and Odoreu) (Figure 3). We can also see that mosquito activity likely already started prior to the start of the experiment due to the high numbers already observed in week 24. Activity gradually declined, particularly in the Tulcea region, until there were only more or less sporadic catches left after week 36.

### 3.1. Mosquito species feeding on pigs

Of the 356 mosquito (and 1 tabanid) samples that we successfully identified, a total of 130 showed visual traces of blood meals (Table 1). Of these, 116 (89.2%) tested positive for porcine blood (Cq values from 22.5 to 34.8 in the Suid assay). Upon testing for the presence of mammalian blood in the remaining 14 mosquito samples, one *Aedes caspius* caught in Sălcioara tested positive for bovine blood (*Bos taurus*) (Cq-value of 29 in the mammalian assay,). The remaining 13 samples tested negative for any mammalian blood in the analysis, despite the visual trace of blood. The one horsefly tested negative for any blood.

**Table 1:** Overview of the number of mosquitoes and horseflies (*N*) caught in the pig cages of each species in total and per location, as well as the number (Npos) and percentage (%pos) of porcine-blood PCR positive samples per species.

						**Location**															
						**Satu Mare**								**Tulcea**							
		**Total**				**Ambud**				**Odoreu**				**Sălcioara**				**Somova**			
**Genus**	**Species**	***N***	**Npos**	**%pos**	Σ**pos**	***N***	**Npos**	**%pos**	Σ**pos**	***N***	**Npos**	**%pos**	Σ**pos**	***N***	**Npos**	**%pos**	Σ**pos**	***N***	**Npos**	**%pos**	Σ**pos**
*Aedes*	*caspius*	24	8	33.3	6.9	0	0	0	0	0	0	0	0	15	8	53.3	47.1	9	0	0	0
	*flavescens*	1	1	100	0,9	0	0	0	0	0	0	0	0	1	1	100	5.9	0	0	0	0
	*geniculatus*	25	12	48	10.3	11	6	54.5	9.2	13	5	38.5	17.2	1	1	100	5.9	0	0	0	0
	*riparius*	1	0	0	0	0	0	0	0	0	0	0	0	1	0	0	0	0	0	0	0
	*vexans*	20	18	90	15.5	16	15	93.8	23.1	4	3	75	10.3	0	0	0	0	0	0	0	0

*Anopheles*	*hyrcanus*	4	2	50	1.7	0	0	0	0	0	0	0	0	3	2	66.7	11.8	1	0	0	0
	*maculipennis s.l*.	15	12	80	10.3	4	4	100	6.2	2	2	100	6.9	2	1	50	5.9	7	5	71.4	100
	*plumbeus*	2	1	50	0.9	1	1	100	1.5	0	0	0	0	1	0	0	0	0	0	0	0

*Culex*	*modestus*	4	2	50	1.7	0	0	0	0	4	2	50	6,9	0	0	0	0	0	0	0	0
	*pipiens/ torrentium*	236	44	18.6	37.9	169	37	21.9	56.9	53	3	5.7	10.3	14	4	28.6	23.5	0	0	0	0

*Culiseta*	*annulata*	22	16	72.7	13.8	3	2	66.7	3.1	19	14	73.7	48.3	0	0	0	0	0	0	0	0
	*longiareolata*	1	0	0	0	0	0	0	0	1	0	0	0	0	0	0	0	0	0	0	0
	*morsitans*	1	0	0	0	0	0	0	0	0	0	0	0	0	0	0	0	1	0	0	0

*Haematopota*	*pluvialis/ subcylindrica*	1	0	0	0	0	0	0	0	0	0	0	0	1	0	0	0	0	0	0	0
Total		357	116	32.5	100	204	65	31.9	100	96	29	30.2	100	39	17	43.6	100	18	5	27.8	100

Σpos indicates the percentage that each species contributes to the total number of porcine-blood PCR positive samples, in total as well as per location. Mosquitoes without visual blood in abdomen (Sella score of 1 and 7) have been excluded from PCR-analysis. The *Haematopota pluvialis/subcylindrica* could not be Sella scored, but was still included for PCR-analysis.

While only 18.6% of the collected *Culex pipiens/torrentium* carried a porcine blood meal (44 out 236), this species still constituted the largest number of mosquitoes carrying pig blood due to their high abundance (44 positive *Culex pipiens/torrentium* out of 116 positive mosquitoes in total, or 37.9%) (Table 1). Among the other species of which we caught at least five specimen, *Aedes vexans* (18 out of 20, i.e. 90 or 15.5% of all porcine blood meals), *Anopheles maculipennis* (12 out of 15, i.e. 80 or 10.3% of all porcine blood meals) and *Culiseta annulata* (16 out of 22, i.e. 72.7 or 13.8% of all porcine blood meals) showed the highest proportions feeding on domestic pig. These four species combined comprised 77.5% of all porcine blood meals. Other (non-sporadic, i.e. >5) species were *Aedes geniculatus* (12 out of 25, i.e. 48 or 10.3% of all porcine blood meals), and *Aedes caspius* (8 out of 24, i.e. 33.3 or 6.9% of all porcine blood meals). Note that *Aedes caspius* was only caught in cages from the Tulcea region.

### 3.2. Seasonality vs. proportion of mosquitoes with pig blood

**Figure 4:**
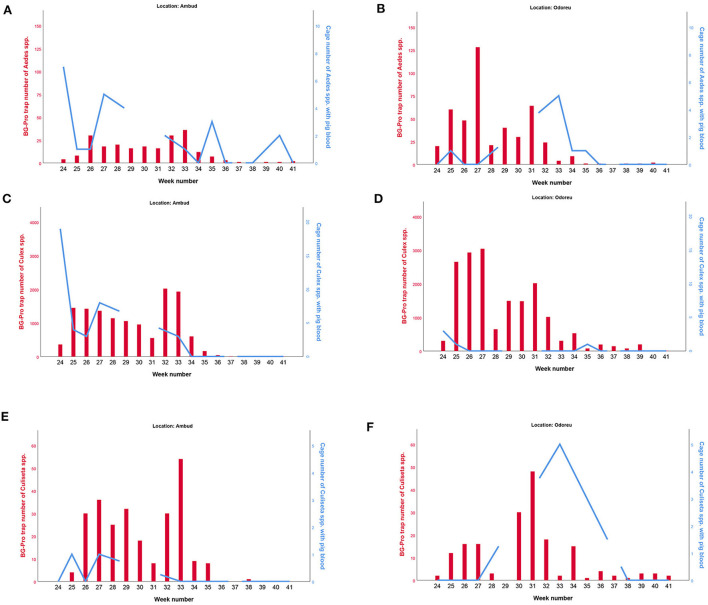
Seasonal abundance of the different genera of mosquitoes caught in our BG traps (red bars and the left Y-axis), as well as the number of mosquitoes with pig blood of the specific mosquito genera in our cages (blue lines and the right Y-axis). *Aedes spp*. is shown in **(A,B)**, *Culex spp*. is shown in **(C,D)**, *Culiseta spp*. is shown in **(E,F)**. Ambud is shown in the left **(A,C,E)** and Odoreu is shown in the right **(B,D,F)**, both locations are situated in northwestern Romania. Note that the proportion of mosquitoes with pig blood are based on the sum of all the individual species caught for each genera, and only porcine-blood PCR-positive cases are included. Left and right Y-axes scales are not identical, and axes scales between the different genera are not identical. Anopheles are not included as we had too few observations to make any meaningful plots.

We plotted the overall mosquito seasonal abundance data from our subsampled BG-Pro trap collections for each genera together with the number of mosquitoes with pig blood in the cages (Figure 4). *Culex* spp. was significantly more abundant than the other genera. Collections tended to be more numerous in Odoreu compared to Ambud for all genera. Overall, vector seasonal abundance increased the first 2 weeks from the onset of our BG-Pro trap collections and decreased towards the end of the collection period to almost zero. While peak activity in Ambud occurred in week 32 and 33 for all genera, both *Aedes* spp. and *Culex* spp. activity peaked in week 27 in Odoreu, while *Culiseta* spp. peak activity was later in week 31.

For all four mosquito genera and for both Ambud and Odoreu, we tested for a correlation between the seasonal abundance (i.e. BG-Pro trap results) and the proportion of blood fed mosquitoes of that genus in our cages using Spearman's correlation test. None of these tests found a significant correlation between seasonal abundance and the proportion of mosquitoes having fed on pigs.

## 4. Discussion

Of the 356 successfully identified mosquitoes caught in the cages, 116 carried a porcine blood meal, while one *Aedes caspius* in Sălcioara carried a bovine blood meal (*Bos taurus*) and the remaining 239 did not carry any identifiable blood meals. The bovine blood meal finding indicates that blood from different hosts is occasionally introduced to backyard pig farms and that blood fed mosquitoes are still attracted to backyard pigs or their enclosures. Given this finding, we argue that a mosquito carrying an ASFV blood meal, obtained from a nearby infected domestic pig or wild boar, could potentially introduce ASFV to a farm. To cause an outbreak, virus transmission from the mosquito to domestic pigs is needed, for example by the mosquito taking a consecutive blood meal from the backyard pig, as has been shown possible in previous studies using *S. calcitrans* (23). Additional modes of transmission could for example be through the pig ingesting the mosquito, as has been shown sufficient for *S. calcitrans* (21), or environmental contamination of the pig enclosure. However, there are significant differences in the volume of a blood meal taken between potential vectors. *S. calcitrans* is known to take blood meals between 11.2 and 15.1 μl (35), while horseflies can take blood meals ranging from 20 μl (*Chrysops* spp.) to almost 700 μl (*Tabanus attratus*) (36), as they can consume more than their own weight in blood (37). Mosquito blood meals are significantly smaller, ranging, for example, between 1.3 μl and 5.4 μl in *Anopheles stephensi* (38), or 1.86–3.74 μl in *Aedes aegypti* (39). Previous studies have identified close proximity to outbreaks in domestic farms to be a risk factor for ASF occurrence in Romanian farms (27). Our findings support that mosquitoes do bring exogenous blood meals in close contact with backyard pigs.

Some mosquitoes that we caught inside the cages, are species known to be predominantly ornithophilic (i.e. *Culiseta longiareolata* and *Culiseta morsitans*) (Appendix I). These mosquitoes did not show any signs of blood meals. Conversely, some mosquitoes that we caught inside the cages are species known to be primarily ornithophilic (i.e. *Culex pipiens/torrentium* and *Culiseta annulata*) (Appendix I). Some of these mosquitoes from the pig cages did carry visual blood meals but tested negative for both porcine and mammalian blood. Catching these mosquitoes in the cages indicates that the predominantly ornithophilic mosquitoes are still attracted to the pigs or their enclosures, despite not feeding on the pigs. It also shows that the occasional ornithophilic species, despite already carrying a (non-mammalian) blood meal, are attracted to the pigs or their enclosures. *Culex pipiens* could therefore have taken a WNV or JEV viraemic blood meal before coming into contact with domestic pigs. The finding of these mosquito species next to pigs suggests that these mosquitoes may introduce JEV to pig stables without being biological vectors, which we argue could potentially still transmit the virus either by being ingested by a pig or through environmental contamination.

Despite having one of the lowest proportions of porcine-blood meals in the cages, the predominantly ornithophilic *C. pipiens/torrentium* still accounted for the highest number of the identified porcine blood meals (44 out of 116). This is due to their higher abundance compared to the other species caught (236 out of 356). Kilpatrick et al. (14) coined this phenomenon the Bridge Vector Paradigm. This paradigm describes the phenomenon where a potential vector is deemed unimportant for a particular pathogen from a qualitative perspective, conversely becomes a key vector species when studied in a quantitative manner. Their findings show that *Culex pipiens* and *Culex restuans*, two species previously considered unimportant in humans infections due to their predominantly ornithophilic feeding behaviour, are actually responsible for up to 80% of WNV infections in humans in the northeast United States due to their abundance compensating for the proportional feeding preference for mammalian blood. We argue that, assuming random selection of host species between individual *C. pipiens/torrentium* mosquitoes, the vast majority of *C. pipiens/torrentium* that take a second or third blood meals will at some point likely have fed on bird blood, which in turn could make them important bridge vectors for WNV and JEV. However, the abundance of *C. pipiens/torrentium* could be exacerbated due to the (semi-) urban settings we used for our collection, as *C. pipiens* is known to prefer (semi-) urban habitats over rural/wild habitats (40).

The use of *C. pipiens/torrentium* in this study, which is a combination of the *C. pipiens* complex (i.e. *C. pipiens pipiens* and *C. pipiens molestus*) and *C. torrentium*, was due to the females of these species/biotypes being very difficult to reliably distinguish (16). This however limits the conclusions that can be drawn from the study, as *C. pipiens pipiens* and *C. pipiens molestus* exhibit different feeding behaviours, with *C. pipiens pipiens* being primarily ornithophilic and *C. pipiens molestus* feeding on both birds and mammals (41), with humans representing 20% of blood meal hosts of *C. pipiens molestus* in Romania (42). Because of this difference, *C. pipiens pipiens* are believed to play only a minor role in the spread of arboviruses in Europe (16), although *C. pipiens* (in combination with *C. restuans*) are reported to account for over 80% of WNV infections in the north-eastern United States (14). As for *C. pipiens* and *C. torrentium, C. pipiens* is reported to be the dominant species in southern Europe, with *C. torrentium* rarely being reported. In northern Europe, *C. torrentium* is the dominant species while in central Europe similar proportions of *C. pipiens* and *C. torrentium* are observed (41). Nicolescu reported that, in Romania, *C. pipiens s.l*. is more abundant in urban areas compared to *C. pipiens molestus*, while in rural areas, they are abundant in animal shelters along with *Anopheles* spp. (43). Tiron et al. (42) however reported that, in southeastern Romania, *C. pipiens pipiens* has a preference for “green areas,” while *C. pipiens molestus* prefers human settings and animal farmland. Given the location of our study sites (i.e. animal shelters in rural southeastern Europe) and the described differences in feeding behaviour, the blood fed *C. pipiens/torrentium* in our study (44 out of 236 contained pig blood) are likely to *C. pipiens molestus*, although we cannot state this for certain. *C. pipiens* is considered the main vector of WNV in southern Romania, while *Anopheles* spp. could also play a role in rural environments (43). *Aedes vexans*, the second-most frequent feeder on pig blood caught in the cages (18 out of 20 contained pig blood), is a predominantly mammalophilic species most abundant around floodplains or lakes (16, 44), that can take multiple blood meals (16). *Culiseta annulata*, the third-most frequent feeder on pig blood caught in the cages, also displays a strong preference for mammals (16) and frequently enter animal enclosures or houses to feed on humans or domestic animals during the summer months (16).

While the vast majority of mosquitoes with visual signs of a blood meal also tested positive for porcine blood (89.1%), 14 samples (10.9%) did not. Besides one sample with *Bos taurus* blood, we were unable to state for certain that the remaining 13 samples did not carry blood of mammalian origin. Most likely, the blood meals within the remaining 13 samples were too degraded, the amount of blood within the samples were too low, or they were of non-mammalian origin.

We did not observe any clear correlation between the proportion of mosquitoes with pig blood (i.e. pig blood fed mosquitoes in the cages) and seasonal abundance (i.e. BG-Pro trap mosquitoes) of the various mosquito genera we caught (Figure 4). Throughout the sampling period, *Culex* spp. were significantly more abundant in the BG-Pro traps than the other genera ($\bar{x}$ = 92.3%, max = 99.2%, min = 60%). Other studies on mosquito abundance also indicate that *Culex* is the most abundant mosquito genus throughout Europe, particularly *Culex pipiens* (28).

We observed two seasonal peaks in *Culex* spp. (Figure 4) in both locations (Ambud and Odoreu). Although we cannot say what the vector activity was prior to week 24, personal communication with people at both locations tells us mosquito activity was very low before the onset of our collection period. Interestingly, for both *Aedes* spp. and *Culex* spp., the cage collections with the highest number of mosquitoes with pig blood occurred in the first week of the experiment (week 24). This was several weeks before the observed seasonal peak of these two genera in the BG-Pro traps (week 32 + 33). Furthermore, personal communication also tells us that the region was sprayed with insecticide from a helicopter right after our collection in week 27, from which it appears we can see the effect particularly in Odoreu. While the most abundant species of the *Culex* genus, *Culex pipiens*, is known to go through up to 7 generations in a single year (16), the insecticide sprayed over the region by helicopter following the collection in week 27 could also potentially explain the apparent double peaks.

Since insects were collected once every 24 h, after we fitted the roof on the cages, any mosquito taking a blood meal directly after the roof placement would have up to 24 h to digest the blood meal before collection. According to the digestion times given by Detinova et al. (31), a mosquito could digest enough blood within these 24 h to reach a Sella score of up to 4 (i.e. practically half full and half empty with dark blood). Interestingly, 25 out of 28 samples with a Sella score of 5, and 7 out of 9 with a Sella score of 6 tested positive for pig blood, which indicates that these blood meals either came from other pigs in the vicinity or were only partial/interrupted blood meals. It should be noted however, that the rate of blood meal digestion in mosquitoes, being poikilotherms, varies according to the temperature in the surrounding environment (45). We initially assumed that a higher Sella score (i.e. the blood meal being in a later state of digestion) would correlate to higher Cq-values for our PCR assays. However, a scatterplot comparing Cq-values to Sella scores provided an *R*^2^ correlation coefficient of 0.0004, indicating a negligible correlation between the two variables (data not shown).

Our findings contribute to our understanding of which mosquito species are relevant when studying vector-borne pathogens that involve pigs. *Culex pipiens*, despite not preferentially feeding on pigs, should be the mosquito species of primary focus when looking at mosquito-borne pathogens, due to its high abundance. Other mosquito species, that do not tend to feed on pigs, could still be relevant vectors in terms of the introduction of viraemic blood from other host species, such WNV, as these ornithophilic mosquito species are still attracted to pigs or their enclosures. Future studies are needed to assess, which specific pathogens are introduced by mosquitoes to backyard pigs or their enclosures, and in what quantities. It is also important to gain more insights into the flight distance of these blood fed mosquitoes, to understand the possible range of transmission from viraemic blood-carrying vectors.

## Data availability statement

The raw data supporting the conclusions of this article will be made available by the authors, without undue reservation.

## Ethics statement

The animal study was reviewed and approved by Bioethics Commission of USAMV Cluj-Napoca.

## Author contributions

JS, AM, RB, LK, and AB: conceptualisation. JS, AM, OB, MM, and VA: field data collection. JS, AO, and TR: sample processing. JS: data analysis and draft writing. JS, RB, AM, AO, LK, AB, TR, MM, and VA: draft review. All authors contributed to the article and approved the submitted version.
