# Supplementary material for: Potential mosquito vector attraction to- and feeding preferences for pigs in Romanian backyard farms
Source: Front Vet Sci. 2023 Jan 4;9:1046263. doi: 10.3389/fvets.2022.1046263 (PMC9846066; doi:10.3389/fvets.2022.1046263)
Supplement: Supplementary file 1 [file Data_Sheet_1.PDF]

Appendix I:

We provide a summary of each of the mosquito species caught, as well as the number caught and the number and percentage of mosquitos with pig blood for each species, as well as genus. We also give a brief description of their relevant feeding behaviour, distribution in Europe as well as known or suspected vector potential.

Aedes (N = 73, 41 (56.2%) porcine-blood PCR positive)
Aedes caspius (8 out of 24, or 33.3% porcine-blood PCR positive)<br>Coastal species occurs throughout most of Europe. Goes through several generations per year. Feeds both day and night when abundant, while predominantly crepuscular when less abundant and known to feed on humans. Known vector of the causative agent of tularaemia. [(1)]
Aedes flavescens (1 out of 1, or 100% porcine-blood PCR positive)<br>Wide distribution throughout Europe, apart for some Mediterranean countries, frequents reed beds and can tolerate brackish water. Occurs in low numbers and feeds during crepuscular hours on large mammals. Considered unimportant as a vector due to its low abundance. [(2)]
Aedes geniculatus (13 out of 26, 50% porcine-blood PCR positive)<br>Wide distribution throughout Europe, but most abundant in south-eastern Europe where females bite during the day [(3)]. Experimentally shown to be a potential vector of yellow fever and chikungunya [(2)].
Aedes riparius (0 out of 1, or 0% porcine-blood PCR positive)<br>Uncommon species, mainly found in woodland. Tends to feed on humans, but occurs in such low numbers that it is considered unimportant as a vector. [(2)]
Aedes vexans (19 out of 21, 90.5% porcine-blood PCR positive)<br>Wide distribution throughout Europe. Active during spring and summer, periodically in very high numbers. Population pressure following mass emergence is known to result in migration of at least 15 km, and occasionally multiple times that distance. Known vector of Rift Valley fever virus, potential vector of West Nile virus. Mammalophilic, with a strong anthropophilic tendency, as well as other domestic mammals. [(4)]
Anopheles (N = 21, 15 (71.4%) porcine-blood PCR positive)
Anopheles hyrcanus (2 out of 4, or 50% porcine-blood PCR positive)<br>Wide distribution through southern and central Europe. Emerges around late April or May, with the population size increasing towards to autumn. Common in stables or shelters. Mainly vespertine and nocturnal biting behaviour, focusing on livestock but also humans. Potential vector of malaria. [(4)]
Anopheles maculipennis s.l. (12 out of 15, or 80% porcine-blood PCR positive)<br>This complex of species prefers calm waterbodies, with a wide distribution throughout Europe. Tends to feed on livestock but also feeds on humans when the former is not available. [(2)] One of the primary vectors of malaria. [(5)]

Anopheles plumbeus (1 out of 2, or 50% porcine-blood PCR positive)<br>Found in Europe from late spring until the end of September. Aggressive biter, feeding at any time of the day of different mammalian hosts, birds and reptiles, while some population show strong anthropophilic behaviour. Considered an effective carrier of malaria but thought to be of minor importance due its ecology. Because of its ornithophilic and anthropophilic behaviour, *Anopheles plumbeus* is considered a potential bridge vector for the transmission of West Nile Virus from birds to human and has experimentally been shown to be a potential vector of WNV. (6)
Culex (N = 252, 48 (19%) porcine-blood PCR positive)
Culex modestus (2 out of 4, or 50% porcine-blood PCR positive)<br>Aggressive biter known to feed on a wide variety of vertebrates, including birds and humans. Primarily feeds outdoors during evening hours. Wide distribution throughout Europe, except for Finland, Norway and the Baltic states. Potential vector of WNV and Usutu Virus. (4, 7)
Culex pipiens/torrentium: (46 out of 248, or 18.5% porcine-blood PCR positive)<br>Wide distribution throughout Europe, often very abundant. Prefers (semi-) urban habitats over rural/wild habitats [8]. Feeds at night on a wide range of vertebrate hosts, including birds and humans. Moves into stables during the day to rest. Major vector of WNV and Usutu Virus, as well as several other arboviruses. Potential vector of RVFV and JEV. Also known to act as a vector for avian malaria and filarial worms. (9)
Culiseta (N = 25, 18 (72%) porcine-blood PCR positive)
Culiseta annulata (18 out of 22, 81.8% porcine-blood PCR positive)<br>Aggressive biter in spring, feeding on both humans, mammals and occasionally birds, during the day. A shift in feeding behaviour is observed in the summer months where they feed more nocturnally indoors on both humans and domestic animals. (4)
Culiseta longiareolata (0 out of 2, or 0% porcine-blood PCR positive)<br>Distribution focused mostly around southern Europe. Predominantly ornithophilic species and considered to be vectors of blood parasites in birds. [2, 4]
Culiseta morsitans (0 out of 1, 0% porcine-blood PCR positive)<br>Wide distribution throughout Europe. Primarily ornithophilic species, but occasionally feeds on small mammals or reptiles. Rarely feeds on humans or large mammals. Considered a potential vector of Eastern equine encephalitis virus. (4)

References

1. Walter Reed Biosystematics Unit (WRBU). *Aedes caspius* species page. Walter Reed Biosystematics Unit Website. http://wrbu.si.edu/vectorspecies/mosquitoes/caspius (2022) [Accessed August 18, 2022].

2. Lindström A & Eklöf D. Stickmyggor i Nordeuropa - en fälthandbok: en illustrerad handbok till stickmyggor i norra och nordvästra Europa. Entomol. Föreningen i Stock. (2022).

3. Walter Reed Biosystematics Unit (WRBU). *Aedes geniculatus* species page. Walter Reed Biosystematics Unit Website. http://wrbu.si.edu/vectorspecies/mosquitoes/geniculatus (2014) [Accessed August 18, 2022].

4. Becker N, Petric D, Zgomba M, Boase C, Madon M, Dahl C, et al. Mosquitoes and their control. British Medical Journal vol. 2 (2010). doi:10.1136/bmj.1.4184.408.

5. Kampen H, Schäfer M, Zielke DE & Walther D. The *Anopheles maculipennis* complex (Diptera: Culicidae) in Germany: an update following recent monitoring activities. Parasitol. Res. 115, 3281–3294 (2016). doi:10.1007/s00436-016-5189-9

6. ECDC, *Anopheles plumbeus* - Factsheet for experts. https://www.ecdc.europa.eu/en/disease-vectors/facts/mosquito-factsheets/anopheles-plumbeus (2022) [Accessed August 17, 2022].

7. Wang L, Rosas ALR, De Coninck L, Shi C, Bouckaert J, Matthijnssens K, et al. Establishment of *Culex modestus* in Belgium and a glance into the virome of Belgian mosquito species . mSphere 6, (2021). doi.10.1128/msphere.01229-20

8. González, MA, Prosser SW, Hernández-Triana LM, Alarcón-Elbel PD, Gior F, López S, et al. Avian Feeding Preferences of *Culex pipiens* and *Culiseta spp.* Along an Urban-to-Wild Gradient in Northern Spain. *Front. Ecol. Evol.* **8**, (2020). doi:10.3389/fevo.2020.568835

9. ECDC, *Culex pipiens* - Factsheet for experts. https://www.ecdc.europa.eu/en/all-topics-z/disease-vectors/facts/mosquito-factsheets/culex-pipiens-factsheet-experts (2020) [Accessed August 17, 2022].
